# Varieties of simulation experience

**DOI:** 10.1186/s41077-019-0115-8

**Published:** 2019-12-20

**Authors:** Neil Tuttle, Gary Grant

**Affiliations:** 1 School of Allied Health Sciences, Griffith University, Gold Coast Campus, Gold Coast, Queensland 4222 Australia; 2 School of Pharmacy, Griffith University, Gold Coast Campus, Gold Coast, Queensland 4222 Australia

In an era of evidence-based medicine, case reports are still considered to *pave the way* for the higher levels of medical research [1] resulting in a number of journals including or being dedicated to publishing case reports. The era of evidence-based education began in the same decade as evidence-based medicine [2], and its application to simulation in healthcare education was advanced by reporting guidelines published in this journal [3]. In contrast to the large number of outlets for case studies in medical journals, there are few outlets for analogous studies in simulation or healthcare education. The *Innovation* article type in *Advances in Simulation* is an exception, and the articles in this supplement describes what are essentially a series of case studies on the use of simulation in allied health education.

The title *Varieties of Simulation Experience* alludes to the seminal 1917 work *Varieties of Religious Experience* by William James [4] in which he states that “a large acquaintance with particulars often makes us wiser than the possession of abstract formulas, however deep.” Simulation in healthcare is a broad church [5], and the articles in this issue take James’ advice by putting an emphasis on the particulars and the experiences with less focus on abstract formulations.

The issue includes a series of stories illustrating some of what developed from the intense activity in simulation that occurred between 2012 and the present in Australia. During this time, the Australian Government through the now disestablished Health Workforce Australia provided over AU$100 million for a range of projects that used simulation to support health professional development. The articles in this supplement represent a small sample of projects that were either directly funded through this initiative or developed from funded projects. Importantly, each of these projects is not requiring ongoing external funding.

The articles are structured as scholarly manuscripts, each including a component of evaluation and each telling a story—or at least part of a story since none yet have an ending. Individually and collectively, it is hoped that the stories acquaint the reader with some *particulars* of simulation experiences, not presuming to make the reader wiser or provide a template, but rather to provide stimuli for their own creativity. Although we believe there is an inherent value in each project, the primary aim in publishing these articles is the potential utility for others in developing their own simulation stories.

The projects have some similarities in that all are patient-centered involving live simulated patients and all involve entry level students and at least one allied health profession. In some ways, the projects are also characterised by their diversity. Ten disciplines are represented, some of which have limited the presence in simulation literature—such as exercise physiology [6–8], nutrition and dietetics [8], aboriginal health liaison, and allied health assistants [6]. Interprofessional learning was addressed by live interactions between combinations of disciplines in some studies [6, 8] and through targeted simulated interactions in others [9, 10]. Innovative uses of technology through video-assisted debriefing [6], simulations via videoconferencing [8, 11], and the integration of an online adaptive learning platform to augment live simulation experiences [10]. While the technological innovations were promising, in each instance, the technology also presented significant challenges.

The duration of the experiences ranged from one [6, 8, 11] to five half-days [10]. The varied aims included discovering the needs of a range of stakeholders [10], preparation for specific aspects [7], settings [6, 8, 10] or areas of practice [8, 10, 11], and general or interprofessional communication skills [6, 8]. Planning and scenario development were the focus of one study [9], and another provided in-depth qualitative exploration of the experiences of facilitators and students [6].

A similar diversity is present in the approaches to evaluation. Most studies included mixed-methods, but the evaluation described in one was purely qualitative [6] and another purely quantitative [10]. Across the studies, all four of Kirkpatrick’s four levels of evaluation of learning. Level 1, reaction was considered in four studies [6–8, 11]; level 2, learning in four [6–8, 11]; level 3, behavior in one; and level 4, results in one [10].

In summary, even with the limited sample of activities described in this supplement, there is a diversity of (1) disciplines, (2) types of interprofessional learning, (3) uses of technology, (4) duration, and (5) methods of evaluation. Most of the projects were developed and conducted by academic staff in universities, while the activities were hosted and/or conducted in educational and/or clinical facilities. Finally, clinical staff do not always have the same access to literature that is enjoyed by academic staff. In order for the projects in this supplement to be in reach all of those who may be interested, we considered it important for it to be open access. As such, our intentions align closely with the open-access model used in *Advances in Simulation* [12].

## Data Availability

No primary data was used for this article.
